# Earliest Evidence for Social Endogamy in the 9,000-Year-Old-Population of Basta, Jordan

**DOI:** 10.1371/journal.pone.0065649

**Published:** 2013-06-11

**Authors:** Kurt W. Alt, Marion Benz, Wolfgang Müller, Margit E. Berner, Michael Schultz, Tyede H. Schmidt-Schultz, Corina Knipper, Hans-Georg K. Gebel, Hans J. Nissen, Werner Vach

**Affiliations:** 1 Department of Anthropology, Johannes-Gutenberg University, Mainz, Germany; 2 Department of Near Eastern Archaeology, Albert-Ludwigs University, Freiburg, Germany; 3 Department of Earth Sciences, Royal Holloway University of London, Egham, United Kingdom; 4 Department of Anthropology, Museum of Natural History, Vienna, Austria; 5 Department of Anatomy, Georg-August-University, Göttingen, Germany; 6 Department of Biochemistry, Georg-August-University, Göttingen, Germany; 7 Department of Near Eastern Archaeology, Free University, Berlin, Germany; 8 Institute of Medical Biometry and Medical Informatics, University Medical Center, Albert-Ludwigs University, Freiburg, Germany; Bristol University, United Kingdom

## Abstract

The transition from mobile to sedentary life was one of the greatest social challenges of the human past. Yet little is known about the impact of this fundamental change on social interactions amongst early Neolithic communities, which are best recorded in the Near East. The importance of social processes associated with these economic and ecological changes has long been underestimated. However, ethnographic observations demonstrate that generalized reciprocity – such as open access to resources and land – had to be reduced to a circumscribed group before regular farming and herding could be successfully established. Our aim was thus to investigate the role of familial relationships as one possible factor within this process of segregation as recorded directly in the skeletal remains, rather than based on hypothetical correlations such as house types and social units. Here we present the revealing results of the systematically recorded epigenetic characteristics of teeth and skulls of the late Pre-Pottery Neolithic community of Basta in Southern Jordan (Figure S1). Additionally, mobility was reconstructed via a systematic strontium (Sr) isotope analysis of tooth enamel of the Basta individuals. The frequency of congenitally missing maxillary lateral incisors in the 9,000-year-old community of Basta is exceptionally high (35.7%). Genetic studies and a worldwide comparison of the general rate of this dental anomaly in modern and historic populations show that the enhanced frequency can only be explained by close familial relationships akin to endogamy. This is supported by strontium isotope analyses of teeth, indicating a local origin of almost all investigated individuals. Yet, the accompanying archaeological finds document far-reaching economic exchange with neighboring groups and a population density hitherto unparalleled. We thus conclude that endogamy in the early Neolithic village of Basta was not due to geographic isolation or a lack of exogamous mating partners but a socio-cultural choice.

## Introduction

The transition from foraging to farming is one of the most fundamental changes in human history, sustained by innovative economic strategies and accompanied particularly by large-scale alteration in social organization [1], [2], [3], [4]. New social and ethological concepts were necessary to sustain living in permanent farming villages at higher population densities [5]. Open access to resources and flexible sharing networks of mobile hunter-gatherer societies had to be reduced to circumscribed groups. There are several possible ways exclusive groups might have defined themselves: besides social, political or ideological criteria, familial relationships might have become influential or decisive.

However, so far the reconstruction of familial and social changes in the Near East is almost exclusively based on architectural, economic, and archaeological data [3], [4]. Although there is some descriptive evidence for anatomical variants interpreted as familial characteristics [6], [7], [8] systematic studies of anthropological data are still rare [9]. Because of the poor preservation of ancient DNA in Mediterranean climates [10], [11], [12], genetically determined anatomical traits are still the most valuable proxies for examining 'genetic kinship' in (pre-)historic populations from the Fertile Crescent. In the framework of the SIGN-Project (Figure S1, Text S1) we therefore systematically recorded epigenetic characteristics of teeth and skulls from Basta in Southern Jordan. This site provides conclusive results demonstrating the organization of this early Neolithic community.

**Figure 1:**
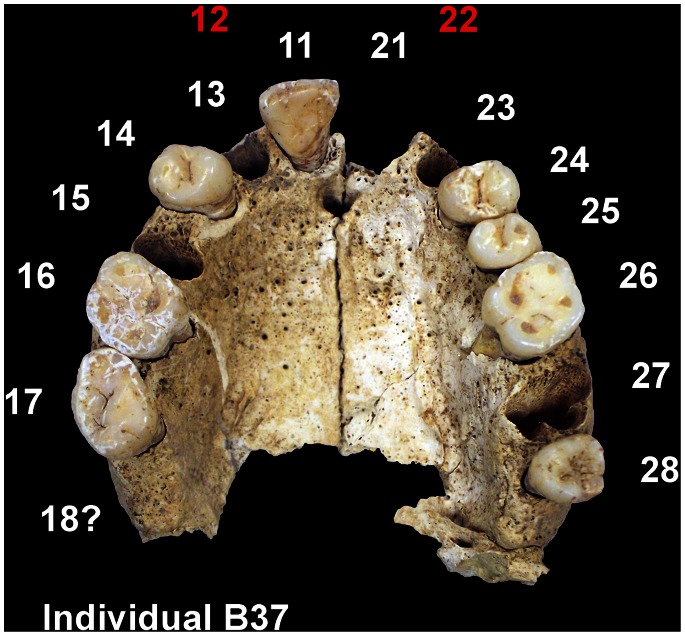
Bilateral maxillary lateral incisor agenesis (MLIA) from the Pre-Pottery Neolithic site of Basta, southern Jordan. The individual B37, a male, is one of ten affected adults (total n = 28). The ^87^Sr/^86^Sr ratios of his first and third molar match the local baseline data and are consistent with growing up at the site. Red: congenitally absent tooth, white: present tooth.

Here we present both dental and isotope geochemical fingerprints to gain insights into both the gene pool and the origin of the Basta inhabitants. The unusually high incidence of a very rare anatomical variant occurring in the population of Basta – namely the agenesis (which means the inherited absence) of maxillary lateral incisors (MLIA), (Figure 1) – is interpreted as an indicator of close genetic relationships, akin to endogamy. The Oxford Dictionaries clearly define the anthropological and biological backgrounds of endogamy [13]. Endogamy is in an anthropological sense “the custom of marrying only within the limits of a local community, clan, or tribe”. In a biological sense it is “the fusion of reproductive cells from related individuals”. This is usually called “inbreeding”, anthropologically a “breed from closely related people or animals, especially over many generations”. It must be emphasized that our analysis gives information exclusively on biological genetic relationships and has nothing to do with familial organization in a socio-cultural sense.

The presence of one key trait which is very rare in a genetically well-mixed population, but very common in Basta leaves little doubt that the individuals came from an endogamic population similar to that observed e.g. by Jöhr [14]. To gain additional independent information on mobility patterns, strontium isotope analyses of tooth enamel were performed.

## Materials and Methods

The early Neolithic site of Basta (30°13′47′′N, 35°32′06′′E) is one of the largest so called mega-sites in southern Jordan [15] (Figures S1–Figure S6, Text S1). Its estimated extent is about 10 ha. Although only three areas (A–C, total: 860 m^2^) were excavated between 1986 and 1992, the findings represent a diversified picture of daily life during the Pre-Pottery Neolithic B (LPPNB) (7500–7000 cal BC). Six radiocarbon samples date the site to between 7540 and 7040 calBC [16].

All necessary excavation permits were obtained for the described field studies from the Department of Antiquities, Amman.

The ecological setting (see Figure S6 for details of the local geology) near a permanent spring, and with access to large herds of wild animals in the nearby steppe environments and to abundant flint deposits, made the site attractive to early Neolithic hunters, farmers, and herders; but the close proximity of dry-steppe environments made rain-fed agriculture a risky enterprise. The vulnerability of the environment was probably high.

The architecture of Basta [15] is very complex with agglutinating buildings and flat roofs that served as space for daily activities and for walking (Figure S3). The slope was leveled by large terraces on which the buildings were constructed. Within the agglomeration of buildings in Area B, several sub-units (“houses”) were discerned, which might provide hints of social organizations; but because excavations stopped on the floor level for conservation reasons, no burials related to these building lots were found. Because of the architectural style and the communal efforts involved in the preparation of the building lots the excavator suggested that late PPNB villages were inhabited by extended families [1]. The identification of separate houses was hardly possible in Area A, but our analysis of epigenetic markers shows that related individuals were not restricted to one part of Area A, or to an isolated domestic unit, but were buried all over the area and in both occupational phases (AIII and AII).

In area A at least three occupational phases were identified (Figure S3). The buildings of the earliest phase (AIII) were constructed directly on residual soil. During the second main building phase (AII) rooms were added and several parts of the buildings enlarged and during the third and last phase (AI) the buildings were filled with rubble, containing flints and other material that had washed down from the slope above.

Raw materials (or finished products of these raw materials) from the area of Petra 15 km to the northwest and from the eastern flanks of the Wadi Araba 30–40 km southwest are thought to have been acquired by the inhabitants of Basta through exchange with other communities [17]. Turquoise from Wadi Maghara in south-west Sinai (250 km) was used for beads. From Timna unworked pieces of green and brown sandstone (Malachite and Limonite) were imported [18] (Figure S5).

In contrast to these imports for decorative items, most of the flint found in Basta was of local origin, only 13% of it being composed of rare and exotic raw materials. Bidirectional blade blanks production was so intensive that it exceeded the local needs and these blanks were probably manufactured for exchange. Basta therefore has been considered “a market center for traded chipped and ground stone artifacts” [19].

Basta’s involvement in a trans-regional exchange network is not only demonstrated by its imported raw materials, but also by the similarities in style of its prestige and decorative items, such as the mother of pearl amulets and the characteristic stone rings, with those of other sites [20].

### Geological Setting of Basta in Southern Jordan

Basta is located in the transitional zone from limestone, dolomite and marl formations in the west to bituminous limestone, marl, chalky marl, and bituminous chalky marl towards the east. About 20 km walking distance north-northwest of the site, the landscape is dominated by the Paleozoic sandstone formations of the Greater Petra Area [17].

Bedrock in the Basta area is dominated by Late Cretaceous limestones, sandy limestones, and dolomites; a calcareous bedrock with quartzite veins was exposed in Area A.

A 10 m deep trench in Area C showed bedded calcarenites and layers of clay and marl. Because slope inclination in Basta exceeds 20% in some places, soil erosion must have been considerable [21]. The variable bedrock geology surrounding the site leads to variable Sr-isotope ratios in food webs (Figure S6) [22], which are key factors in tracing migration.

### Living with the Dead – the Burials of Basta

Most of the burials were discovered in the earliest and the main occupational phases (AIII-AII). In Area A (Figure S3) single or collective, primary and secondary burials as well as isolated skull deposits were recovered inside buildings and in substructure channels. In Area B, only the displaced bones of one individual, some other scattered human bones, and a multiple burial were found. In Area C, very few human remains were discovered. It has been suggested that Area A might have been used as a burial place after the houses had been abandoned [15]. Human remains of Area A were examined for demographic, taphonomic, and pathological features [23]. The authors determined the minimum number of individuals to be 56.

For the present study we analyzed all available individuals with well-preserved upper jaws (n = 28) to determine the presence or absence of maxillary lateral incisors on at least one side (Table 1). All diagnoses were confirmed by X-ray imaging. For Sr-isotope analysis, 32 human enamel samples were available from 22 individuals, including pairs of early vs. late formed enamel of ten individuals. Ten samples of Neolithic animal bones and of the local soil constrain the local Sr-isotope signal.

**Table 1:** Results of all individuals investigated by dental and/or Sr-isotope analysis from Basta.

ID*	Age (yrs)	Sex	Tooth	^87^Sr/^86^Sr	±2 SE	MLIA	Microdontia or other agenesis
B2	20–25	f	16	0.708285	0.000051	(− −)	(− )
			18	0.708180	0.000050		
B4	30–39	f	26	0.708193	0.000010	(− +)	(− )
			28	0.708143	0.000014		
B5	4–8	?	26	0.708170	0.000014	(− −)	(− )
B6/1	30–49	f	18	0.708165	0.000059	(+ −)	(− )
B7/1	10–12	?	26	0.708163	0.000030	(− −)	(− )
			25	0.708137	0.000071		
B7/2	8–12	?	36	0.708177	0.000063	(− −)	(− )
			25	0.708182	0.000012		
B7/3	30–45	f (?)				(− −)	(− )
B7/4	11–14	?	46	0.708163	0.000027	(− −)	12 reduced size
			38	0.708188	0.000035		
B8/1	4–8	?	16	0.707991	0.000040	(− −)	(− )
B8/2	4–5	?				(− −)	(− )
B9	30–39	m (?)	18	0.708164	0.000012	(+ ?)	(− )
B14	40–55	f	26	0.708840	0.000035	(+ −)	(− )
B16/1	45–69	f (?)	48	0.708123	0.000032	(? ?)	(− )
B17	50–59	?				(+ +)	(− )
B20	5–9	?	16	0.708178	0.000039	(− −)	12 reduced size
			24	0.708176	0.000023		
B30	2,5–4	?				(− −)	(− )
B32	5–6	?	16	0.708174	0.000033	(− −)	(− )
B33	13–15	f (?)	36	0.708147	0.000039	(− −)	12 reduced size
B35	22–25	f (?)	36	0.708149	0.000032	(+ −)	35,45 missing
			38	0.708130	0.000031		
B37	30–39	m	36	0.708137	0.000015	(+ +)	28 reduced size
			38	0.708157	0.000014		
B38	40–49	m	48	0.708153	0.000050	(− −)	(− )
B39/1	6–10	?	26	0.708159	0.000075	(− −)	(− )
B39/2	7–10	?	36	0.708499	0.000025	(− −)	(− )
			35	0.708191	0.000015		
B39/3o.4	35–49	m	18	0.708162	0.000014	(+ −)	(− )
B40	50–65	f (?)	18	0.708175	0.000019	(+ +)	(− )
B5060/1	8–11	?	26	0.708066	0.000035	(− −)	22 reduced size
			15	0.708117	0.000028		
B5041	30–39	f (?)				(? +)	(− )
SF7/3083	30–45	m				(− ?)	(− )
SF22/18078/14443	13–17	?				(− ?)	(− )

The age and sex determination of the individuals are based on international standardized methods [23]. The denotation of the teeth follows the two-digit-system of the World Dental Federation. The investigated key feature, the maxillary lateral incisor agenesis (MLIA), has three possible outcomes: presence (+), absence (–), or the trait is indiscernible (?) because the upper jaw itself is missing. The column ‘microdontia or other agenesis’ comprises maxillary lateral incisors with reduced size and/or other types of missing teeth. MLIA and microdontia represent a variable expression of the same developmental defect. Other types of teeth such as the premolars 35 and 45 can additionally be missing [26]. Only three individuals have Sr-isotope values clearly outside the local average ^87^Sr/^86^Sr. Analytical methods for Sr-isotope analysis follows [29] with minor modifications.

### Anthropological Assessment of Genetic Relationships

Non-metric morphological traits (so-called epigenetic/anatomical variants) are a valuable tool for detecting genetically related individuals in (pre-)historic populations if ancient DNA is insufficiently preserved. The method employed includes non-metric cranial and especially dental traits, and has already been tested on many (pre-)historic burial sites. At the basis of identifying groups of genetically related individuals in past populations is the assumption that families share a number of characteristic and specific phenotypical traits. Traits used for kinship analysis must fulfill basic requirements: they must be determined mainly by genetic factors, they must be rare in the general population and the individual traits must be genetically independent from each other. Alt [24] has catalogued more than 100 basic traits suitable for use in kinship reconstruction and inter-population comparisons. The list consists of four sub-groups of traits: anatomical variants of tooth crowns and roots; ontogenetic disturbances of the shape, size, number, structure, and position of teeth; selected non-metric traits of the cranium and jaw; and congenical malformations and syndromes involving jaws and teeth. For the majority of these traits a macroscopic investigation suffices; few need to be diagnosed by X-ray or micro-CT.

The first step in the process of kinship reconstruction is the scoring of traits. In an archaeological context, the preservation of the skeletal material usually prevents the registration of all traits in every individual from a burial site; but if traits are observable the individual is included in the analysis. Most traits are expressed bilaterally, and some traits occur asymmetrically. In most cases, each trait can either be present (+), absent (–), or indiscernible (?), i.e. if the specific skeletal part is missing or insufficiently preserved (e.g. if the occlusal crown surface shows attrition). Some traits are not scored by exclusive alternatives only, such as the fissure pattern of the occlusal surface or the number of tooth cusps and roots. In these cases, the corresponding variant or the actual number of cusps or roots is recorded. Because of the occurrence of different tooth types (incisors, canine, premolar, molar) and the number of teeth (n = 32), the number of the basic traits increases to about 1000 entries per individual [24].

Depending on the structure and size of the skeletal population considered different statistical approaches can be used to identify candidates for subgroups of individuals with genetic relations. Similarity indices for pairs of individuals and statistical methods of searching for larger groups of individuals with a common profile of phenotypes allow the discerning of groups of genetically related individuals in larger populations. Successful applications of this type of analyses can be found in [25]. However, in small skeletal populations like the current one, the comparison of phenotype frequencies with those from general populations is the most promising approach [26] and has been applied successfully [27], [28].

### Statistical Methods

**Figure 2:**
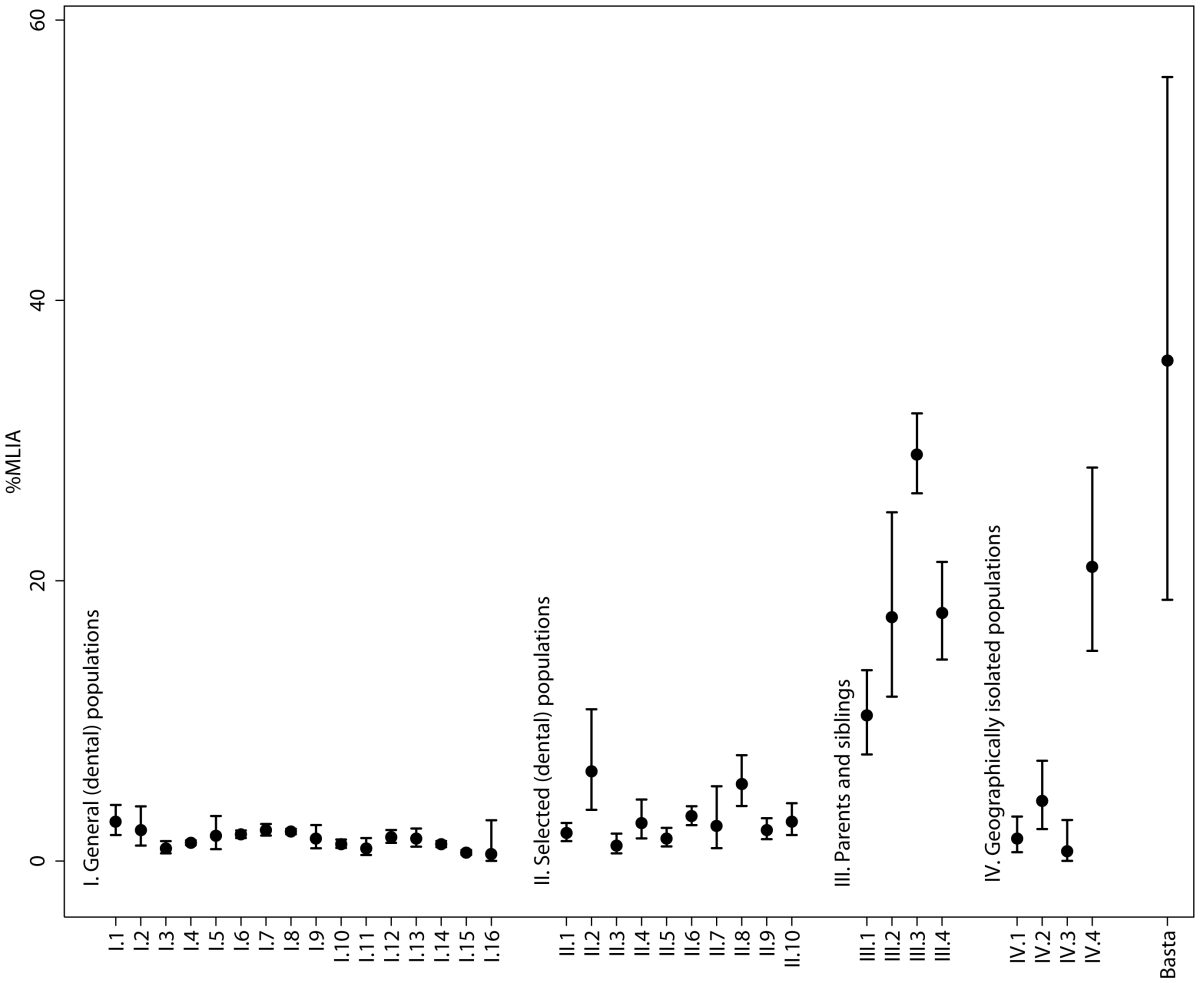
Prevalence (%) of maxillary lateral incisor agenesis (MLIA) for various populations and Basta. I.1–16 Samples from general (dental) populations II.1–10 Samples from selected (dental) populations, III.1–4 Samples from parents and siblings of probands, IV.1–4 Samples from geographically isolated populations. Ranges indicate a 95% confidence interval. References to all studies are given in (Table S1).

In order to interpret the frequency of MLIA in the population of Basta, we compared our results to clinical and genetic population’s studies on the prevalence of MLIA. The p-values comparing the frequency of MLIA (or MLIA and/or microdontia, respectively) are based on an exact binomial two-sided test. The Bonferroni correction was applied by multiplying the p-value by 1,000. This is actually a conservative approach, as among the more than 1,000 traits we studied a substantial number was never discernible in the population of Basta. The confidence intervals in Figure 2 are two-sided exact 95% Clopper-Pearson confidence intervals. The number of subjects with MLIA used in this computation was derived by multiplying the fraction reported in each publication by the overall number of subjects reported, rounding to the nearest integer. All computations were performed in Stata 11.2.

### Strontium (Sr) Isotope Analyses

Powdered tooth enamel samples were taken using dental burrs after removing the outermost surfaces. Sr-isotope analytical protocols follow [29], with the following minor modifications: Prior to dissolution with ∼15 M HNO_3_, enamel powders were leached once using 1 ml of 0.25 M acetic acid to remove the most easily diagenetically-affected Sr. Dissolved samples were spiked with an ^84^Sr tracer, and Sr separated using extraction chromatographic columns using 100 µl SrSpec resin. In view of the small enamel samples (<20 mg) and very low Pb-concentrations (lowest ppb) in earliest Neolithic tooth enamel, no Pb-isotopic analyses were performed. Sr-isotopic compositions and Sr-concentrations were measured using a VG354 thermal-ionization mass-spectrometer (TIMS) at Royal Holloway University of London (RHUL), while several smaller samples were re-analyzed using a TRITON TIMS at The Open University (OU, Milton Keynes, UK), in both dynamic and static Faraday modes, respectively, following loading with a Ta-emitter solution onto zone-refined outgassed Re-filaments. No Sr concentrations are reported because leaching removed non-reproducible amounts of powder for each sample. Analyses of the NIST SRM987 Sr-standard over the course of the measurements yielded 0.710264±0.000015 (2 SD, n = 9; RHUL) and 0.710284±0.000017 (2 SD, n = 2 for one session; OU); consequently OU data were adjusted by −0.00002.

## Results and Discussion

Table 1 summarizes the results of our analyses. In ten adult individuals (35.7% of the total sample) the maxillary lateral incisors were congenitally missing (n = 7 in AIII; n = 3 in AII). MLIA occurs in six females, three males, and one individual of unknown sex. Moreover, four sub-adults have size-reduced incisors, known as microdontia, of which three come from the younger occupation level (AII). This corroborates the overall reduced prevalence of MLIA in the upper layers. MLIA and microdontia represent a variable expression of the same developmental defect, including a negative correlation of both traits [30].

Affected individuals and families can show both different forms of MLIA and microdontia, concerning the degree of affection, variable expressions in between generations, and often an incomplete penetrance. MLIA and microdontia are considered syngenetic features, which is why twins who show MLIA in one individual and microdontia in the other are classified as concordant [31].

In general, tooth agenesis is a well-known hereditary dental anomaly in humans. An evolutionary trend towards a reduced number of permanent teeth has been suggested [32], but systematic analysis of prehistoric human populations for tooth agenesis is missing. Only isolated cases of dental traits have been reported for early Neolithic populations in the Near East [6], [7], [8]. Tooth agenesis has been found to vary remarkably between sexes, and among different populations and tooth types [26]. It is mostly an isolated anomaly; but it can also be associated with oral defects and is part of well-defined inheritable syndromes [33]. Isolated cases of dental agenesis can occur sporadically or familially [34]. Familial-based cases can be the result of a single dominant, recessive, or X-linked gene defect [32]. Numerous concepts about different modes of inheritance have been discussed, such as an autosomal-dominant genetic basis with reduced penetrance and variable expression [35], [36], polygenic inheritance [37], and a threshold model of variation [38]. Although autosomal-recessive inheritance is less probable, it cannot be completely ruled out [39]. The role of *MSX1* and *PAX9* genes in the agenesis of posterior teeth has been demonstrated, but other genes are possible candidates as well, so the role of *MSX1* and *PAX9* for MLIA is contested [33], [40], [41].

In our worldwide comparison of population studies on the prevalence of MLIA, four different population sample types could be identified: I. Samples from general (dental) populations, e.g. unselected patients of dental clinics; II. Samples from selected (dental) populations, e.g. orthodontic patients; III. Samples from parents and siblings of probands, i.e. subjects with known agenesis; and IV. Samples from geographically-isolated populations. These data are summarized in Figure 2 (Table S1) and compared to our results from Basta. In samples from the general dental population, MLIA prevalence ranges between 0.5 and 3%. In samples from selected dental populations we typically observe similar prevalences, which occasionally reach 6%. In the case of relatives of probands, incidence increases to ∼10–30%, reflecting the inheritability of the trait. We identified four geographically-isolated populations. IV.1: In a small village (Schächental, Switzerland) with documented endogamy over several generations, Dietrich [42] observed a (low) agenesis prevalence of 1.6% among 450 investigated inhabitants (of a total of ∼2500). IV.2: An analysis of 1300 inhabitants from a Finnish island showed a prevalence of about 4.3% [30]. IV.3: In a study by Thomsen [43] of 188 descendants of seven women and eight men from the Tristan da Cunha island group (South Atlantic Ocean), the prevalence was 0.7%. IV.4: In a small village (Illgau, Muotatal) in the Swiss Alps, Jöhr [14] examined 162 of approximately 300 inhabitants and found a prevalence of 21.0%. This isolated group experienced probably the highest degree of isolation and endogamy has been well documented there because 77% of all marriages in Illgau over a period of about 230 years were consanguineous: all individuals carrying MLIA can trace their ancestry back to one couple of the early 18th century. Rare traits, as observed in Illgau and Basta, can accumulate in endogamous groups only if they occur already in the founding generation. If they are absent, endogamous structures might be masked (IV.1 [42], IV.3 [43], IV.2 [30], cf. IV.4 [14]).

The extraordinary MLIA prevalence of 35.7% in Basta is significantly higher than in modern non-isolated populations with no more than 3% (see above). This holds true even if we perform a Bonferroni correction to take into account that we have investigated numerous other dental traits in the population of Basta, resulting in a corrected p-value of 4.7×10^−6^. Similarly, the combined rate of MLIA and/or microdontia of 50% in Basta is significantly higher than the maximum rate of 7% in modern populations (p = 1.1×10^−6^). This is incompatible with a straight evolutionary model of increasing tooth agenesis with time, but suggests a combination of genetic and social factors to explain its prevalence [34], [44]. The male:female ratio of 1∶2 in Basta is consistent with the range observed in modern populations worldwide, which on average shows a prevalence of MLIA and/or microdontia 1.37 times higher in females than in males [45].

The increased rate of MLIA in Basta suggests an endogamous mating system for one of the world's earliest farming communities, supporting earlier archaeological hypotheses about endogamy in the early Neolithic elsewhere [4]. Prehistoric endogamy has been suggested for small hunter-gatherers communities or migrating groups due to low population densities [46]. Sholts and colleagues [47] reported an abnormally high prevalence of maxillary canine-first premolar transposition in prehistoric skeletal assemblages from the Santa Barbara Channel Islands of southern California and interpreted these results as evidence for endogamy among these groups.

Evidence for deliberately endogamic historic and modern mating systems has only been reported for urbanized, hierarchical communities, including the Pharaonic dynasties of Egypt, the ruling dynasties of Zoroastrians in Persia, and the Abbad tribe in Jordan [46], [48]. A study of the Iron Age skeletons from the La Tène culture cemetery of Münsingen-Rain (Switzerland) shows a continuous genetic affiliation within the archaeologically identified high ranking group during the entire period the cemetery was in use [25].

In contrast, the evidence of Basta points at a very early case of deliberate segregation by familial relationship, despite a lack in the archaeological records of clearly visible political, economic or ideological status differentiation and hierarchization. The high variability of grave goods and burial rites, the random sex and age distributions of affected individuals, as well as the temporal and spatial distribution of the burials implies a random sample. Endogamy because of status or profession thus seems rather improbable.

But the archaeological data of Basta also suggests that the community was part of a well-developed regional exchange system of raw materials like turquoise, coral, and obsidian (as detailed above, Figure S5). Moreover, similarities in building traditions and material culture also point to Basta’s contacts with other nearby Neolithic groups. Thus the southern Jordanian mountains were no barrier and the endogamous mating system of the early inhabitants of Basta cannot be explained by geographic isolation (Figure S6).

The estimated population size at Basta and the increased population density within the region [49] during the late Pre-Pottery Neolithic B also makes it improbable that a lack of mating partners caused endogamic structures. No correlation of the quantity and/or quality of grave goods, burial rituals or anatomical traits could be discerned. The endogamic mating system of Basta appears to have been a deliberate choice.

One reason for such a restrictive mating system might have been the high vulnerability of the environment and the stress on resources due to increased population densities. Additionally, the beginnings of cultivation and herding required some kind of restricted access to resources. But it is premature to conclude that familial relationships became a general criterion for the more exclusive circumscription of early Neolithic groups everywhere in the Near East [50]. In other communities, ideological or territorial criteria may have been as decisive as familial bonds. Despite some evidence of familial relationships between some individuals at the Pre-Pottery Neolithic sites of Jericho, West Bank, [8] and Abu Hureyra in Syria [6], no such exclusive mating system has been discerned anywhere else so far at that early period.

### Residence Patterns

**Figure 3:**
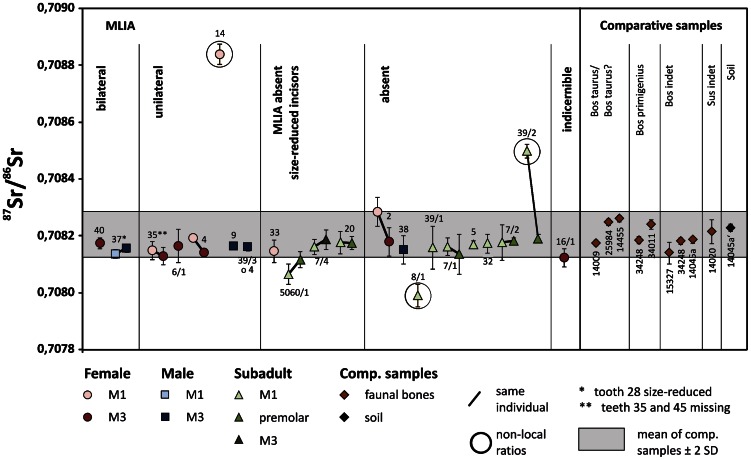
Sr-isotope compositions of tooth enamel (left) and comparative samples (right) from Basta. The former are plotted consecutively and show early and late mineralizing tooth pairs of individuals (highlighted with a tie-line) if applicable. Three individuals (14, 8/1, 39/2) plot clearly outside the ±2 SD local signal for Basta (shaded box: 0.70821±0.00008). Given the reported ^87^Sr/^86^Sr variation between ∼0.7053 and 0.7094 in modern food webs in the region [22], the observed overall variability for Basta is very small. In case of B39/2, only the first molar shows a non-local signature, which indicates that by the time of mineralization of the 2^nd^ premolar (∼year 6), this individual had already moved to Basta. B5060/1 M1 is only marginally below the local Sr-isotope range, which is interpreted not to be significant, also because the Sr-isotope signal of its PM2 is consistent with Basta (even identifying B5060/1 as a non-local would not change any of the conclusions herein).

The endogamous structure at Basta is corroborated by tooth enamel Sr-isotope data, which are remarkably uniform. Nineteen individuals fall within the very narrow range of the local ^87^Sr/^86^Sr signature of 0.70821±0.00008 (2 SD) (Figure 3, Table 2). In contrast to early farming communities in Europe [51], both male and female individuals show local signals, meaning that neither patri- nor matrilocality can be discerned.

**Table 2:** Mean ^87^Sr/^86^Sr ratio and individual data of comparative samples from Basta.

Comparative samples	Species/soil	^87^Sr/^86^Sr	±2 SE
Mean		0.708207	0.000077 (±2 SD)
25984	*Bos taurus*	0.708249	0.000011
15327	*Bos indet*	0.708141	0.000039
34011	*Bos primigenius*	0.708241	0.000016
34248	*Bos primigenius*	0.708186	0.000013
34248[sic!]	*Bos indet*	0.708182	0.000012
14020	*Sus indet*	0.708215	0.000042
14045a'	Soil sample	0.708229	0.000013
14045a	*Bos indet*	0.708187	0.000012
14009	*Bos taurus ?*	0.708176	0.000011
14455	*Bos taurus* ?	0.708262	0.000012

Local animal bones and one soil sample yielded a narrow ‘local’ ^87^Sr/^86^Sr ratio for Basta.

Only three of the 22 individuals had clearly resolvable non-local signals: One child (B39/2) came to Basta before the age of six, as demonstrated by a non-local signal of his first molar but a local signal for the second premolar. An adult woman (B14) with a non-local first molar spent at least the first ∼3.5 years of her life in a region with higher ^87^Sr/^86^Sr ratios. She was affected by MLIA and came to Basta as a child. She was buried in the more recent occupation Phase AII. Different scenarios can be offered to interpret this observation: Her parents might have left Basta for some time, returning with their child when she was around 3.5 years old or the child might have grown up in a nearby familial context. The third individual (B8/1) has a lower Sr-isotope signature. Taken together, the strontium isotope results do not support significant inter-site mobility, even though migration from geologically very similar settings cannot be detected. Further analyses from other ‘mega-sites’ are underway to investigate the hypothesis whether increased inter-side mobility led to the genesis of these large PPNB villages [49].

### Conclusion

Though examples in the more recent past and present are not uncommon, the exceptionally high MLIA incidence within the population of Basta represents the earliest evidence for a self-imposed exclusive mating system. In contrast to earlier hypotheses, our interpretation is based on biological traits combined with isotope geochemical data and thus provides direct and strong evidence for the fundamental social changes that accompanied the transition from mobile hunting-gathering to sedentary farming, during which time flexible social structures were tied into more permanent social bonds.

## Supporting Information

Figure S1 **Map of the studied region.** Location of Epipalaeolithic and early Neolithic sites with human remains investigated in the *SIGN*-Project (map edited by: Felix Schreiber).
(TIF)

Figure S2 **Plan of the modern Basta with the excavated Areas A, B, and C** [**21**] **.**
(TIF)

Figure S3 **Area A.** Architectural remains in Area A (Plan: Basta Joint Archaeological Project/ex oriente e.V.).
(TIF)

Figure S4 **Area B.** Preserved building remains in Area B ('Basta House') (Photo: M. Benz).
(TIF)

Figure S5 **Raw material sources.** Probable sources of raw materials which have been found at the early Neolithic site of Basta [17]. RS = Red Sea area, CJ = Daba, source area of green marble in Central Jordan, ∼50 km south of Amman, GM = Gebel el-Maghara, south-west Sinai (∼250 km southwest of Basta).
(TIF)

Figure S6 **Geological setting of major Pre-Pottery Neolithic sites in southern Jordan.** (Map design: Christoph Purschwitz, by compilation of data from [52]–[58]).
(TIF)

Table S1 **Prevalence of maxillary lateral incisor agenesis (MLIA) within modern sampling groups.**
(DOC)

Text S1 **Supplementary text.**
(DOC)

## References

[1] Gebel HGK (2002) Subsistenzformen, Siedlungsweisen und Prozesse des sozialen Wandels vom akeramischen bis zum keramischen Neolithikum. Grundzüge sozialen Wandels im Neolithikum der südlichen Levante 2. Freiburg: Universitätsbibliothek. Available: http://www.freidok.uni-freiburg.de/volltexte/466. Accessed 2012 Apr 12.

[2] Benz M (2010) Changing landscapes – changing societies? In: Finlayson B, Warren G, editors. Landscapes in Transition. Levant Supplementary Series 8. Oxford: Oxbow. 77–85.

[3] Flannery KV (2002) The origins of the village revisited: from nuclear to extended households. Am Antiq 67: 417–433.

[4] Baird D (2005) The history of settlement and social landscapes in the Early Holocene in the Çatalhöyük Area. In: Hodder I, editor. Çatalhöyük Perspectives. Reports from the 1995–99 Seasons. Cambridge: McDonald Institute. 55–74.

[5] Gebel HGK (2010) Commodification and the formation of early Neolithic social identity. The issues as seen from the southern Jordanian Highlands. In: Benz M, editor. The principle of sharing. Segregation and construction of social identities at the transition from foraging to farming. Studies in Early Near Eastern Production, Subsistence, and Environment 14. Berlin: Ex Oriente. 35–80.

[6] Molleson TI (2000) Appendix 5: the human remains. In: Moore AMT, Hillman GC, Legge JA, editors. Village on the Euphrates. Oxford: Oxford Univ. Press. 533–544.

[7] Belfer-Cohen A (1988) The Natufian graveyard of Hayonim Cave. Paléorient 14: 297–308.

[8] Röhrer-Ertl O (1978) Die Neolithische Revolution im Vorderen Orient. München: R. Oldenbourg Verlag. 324 p.

[9] Pilloud MA, Larsen CS (2011) “Official” and “practical” kin: inferring social and community structure from dental phenotype at Neolithic Çatalhöyük, Turkey. Am J Phys Anthropol 145: 519–530. 21590748 10.1002/ajpa.21520

[10] Bouwman AS, Brown KA, Brown TA, Chilversa ER, Arnotta R, et al (2009) Kinship in Aegean prehistory? Ancient DNA in human bones from mainland Greece and Crete. Annual of the British School at Athens 104: 293–309.

[11] Mahli RS, Van Tuinen M, Mountain J, Hodder I, Hadly EA (2005) Pilot project: Çatalhöyük ancient DNA study. In: British Institute of Archaeology at Ankara Monograph Hodder I, editor. Inhabiting Çatalhöyük - reports from the 1995–1999 seasons. 38: 307–321.

[12] Fernández E, Arroyo E, Pérez-Pérez A, Turbón D (2006) Análisis genético-poblacional des yacimento neolítico de Tell Ramad, Siria. Syria 83: 107–114.

[13] Oxford Dictionaries (2010). Available: http://oxforddictionaries.com/definition/english/endogamy.Accessed 2013 Mar 8.

[14] Jöhr AC (1934) Reduktionserscheinungen an den oberen seitlichen Schneidezähnen – dominant gehäuft in einem Schwyzer Bergdorf. Archiv Julius Klaus-Stiftung 9: 73–131.

[15] Gebel HGK, Nissen HJ, Zaydoon Z, editors (2006) Basta II. The architecture and stratigraphy. Bibliotheca Neolithica Asiae Meridionalis et Occidentalis & Yarmouk Univ., Monograph of the Faculty of Archaeology and Anthropology 5. Berlin: ex oriente. 308 p.

[16] Benz M, editor (2010) PPND – Platform for the publication of Near Eastern radiocarbon data. Available: https://www.exoriente.org/associated_projects/ppnd_php. Accessed 2012 Nov 18.

[17] Hermansen BD (2004) Raw materials of the small finds industries. In: Nissen HJ, Muheisen M, Gebel HGK, editors. Basta I. The human ecology. Bibliotheca Neolithica Asiae Meridionalis et Occidentalis & Yarmouk University, Monograph of the Faculty of Archaeology and Anthropology 4. Berlin: Ex Oriente. 117–128.

[18] Hauptmann A (2004) ‘Greenstones’ from Basta. Their mineralogical composition and possible provenance. In: Nissen HJ, Muheisen M, Gebel HGK, editors. Basta I. The human ecology. Bibliotheca Neolithica Asiae Meridionalis et Occidentalis & Yarmouk University, Monograph of the Faculty of Archaeology and Anthropology 4. Berlin: Ex Oriente. 169–176.

[19] Muheisen M, Qadi N, Gebel HGK (2004) Raw materials of the flint and ground stone industries. In: Nissen HJ, Muheisen M, Gebel HGK, editors. Basta I. The human ecology. Bibliotheca Neolithica Asiae Meridionalis et Occidentalis & Yarmouk University, Monograph of the Faculty of Archaeology and Anthropology 4. Berlin: Ex Oriente. 129–154.

[20] Starck JM (1988) Comparative analysis of stone ring artifacts from Ba‘ja and Basta. In: Garrard A, Gebel HG, editors. The Prehistory of Jordan. The State of Research in 1986. British Archaeological Reports International Series 396, Oxford: British Archaeological Reports. 137–174.

[21] Kamp U (2004) Geomorphological site setting and geochemical results. In: Nissen HJ, Muheisen M, Gebel HGK, editors. Basta I. The human ecology. Bibliotheca Neolithica Asiae Meridionalis et Occidentalis & Yarmouk University, Monograph of the Faculty of Archaeology and Anthropology 4. Berlin: Ex Oriente. 53–94.

[22] Shewan L (2004) Natufian settlement systems and adaptive strategies: the issue of sedentism and the potential of strontium isotope analysis. In: Delage C, editor. The last hunter-gatherer societies in the Near East. British Archaeological Reports International Series 1320. Oxford: Archaeopress. 55–94.

[23] Schultz M, Schmidt-Schultz T, Gresky J, Kreutz K, Berner M (2007) Morbidity and mortality in the late PPNB populations from Basta and Ba'ja (Jordan). In: Faerman M, Horwitz LK, Kahana T, editors. Faces from the past: diachronic patterns in the biology of human populations from the Eastern Mediterranean. British Archaeological Reports International Series 1603. Oxford: Archaeopress. 82–99.

[24] Alt KW (1997) Odontologische Verwandtschaftsanalyse. Individuelle Charakteristika der Zähne in ihrer Bedeutung für Anthropologie, Archäologie und Rechtsmedizin. Stuttgart: Fischer.

[25] Alt KW, Jud P, Müller F, Nicklisch N, Uerpmann A, et al (2005) Biologische Verwandtschaft und soziale Strukturen im Latènezeitlichen Gräberfeld von Münsingen-Rain. Jb RGZM 52: 157–210.

[26] Alt KW, Vach W (1995) Odontologic kinship analysis in skeletal remains: concepts, methods, and results. Forensic Sci Int 74: 99–113. 7665137 10.1016/0379-0738(95)01740-a

[27] Alt KW, Pichler S, Vach W, Huckenbeck W, Stloukal M (1996) Early Bronze Age family burial from Velké Pavlovice. Verification of kinship hypothesis by odontologic and other non-metric traits. Homo 46: 256–266.

[28] Alt KW, Pichler S, Vach W, Klima B, Vlcek E, et al (1997) Twenty-five thousand-year-old triple burial from Dolni Vestonice – an ice age family? Am J Phys Anthrop 102: 123–131. 9034043 10.1002/(SICI)1096-8644(199701)102:1<123::AID-AJPA10>3.0.CO;2-2

[29] Müller W, Fricke H, Halliday AN, McCulloch MT, Wartho JA (2003) Origin and migration of the Alpine Iceman. Science 302: 862–866. 14593178 10.1126/science.1089837

[30] Alvesalo L, Portin P (1969) The inheritance pattern of missing, peg-shaped and strongly mesio-distally reduced upper lateral incisors. Acta Odontol Scand 27: 563–575. 5262405 10.3109/00016356909026309

[31] Schulze C (1987) Anomalien und Mißbildungen der menschlichen Zähne. Berlin: Quintessenz.

[32] Vastardis H (2000) The genetics of human tooth agenesis: new discoveries for understanding dental anomalies. Am J Orthod Dentofacial Orthop 117: 650–55. 10842107

[33] Nieminen P (2009) Genetic basis of tooth agenesis. J Exp Zool B Mol Dev Evol 312B: 320–342. 19219933 10.1002/jez.b.21277

[34] Markovic M (1982) Hypodontia in twins. Swed Dent J Suppl 15: 153–162. 6963769

[35] Arte S, Nieminen P, Apajalahti S, Haavikko K, Thesleff I, et al (2001) Characteristics of incisor-premolar hypodontia in families. J Dent Res 80: 1445–1450. 11437217 10.1177/00220345010800051201

[36] Woolf CM (1971) Missing maxillary lateral incisors: a genetic study. Am J Hum Genet 23: 289–296. 5089845 PMC1706719

[37] Suarez BK, Spence MA (1974) The genetics of hypodontia. J Dent Res 53: 781–785. 4526369 10.1177/00220345740530040201

[38] Brook AH (1984) A unifying aetiological explanation for anomalies of human tooth number and size. Arch Oral Biol 29: 373–378. 6611147 10.1016/0003-9969(84)90163-8

[39] Pinho T, Maciel P, Lemos C, Sousa A (2010) Familial aggregation of maxillary lateral incisor agenesis. J Dent Res 89: 621–625. 20400722 10.1177/0022034510364486

[40] Pinho T, Silva-Fernandes A, Bousbaa H, Maciel P (2010) Mutational analysis of MSX1 and PAX9 genes in Portuguese families with maxillary lateral incisor agenesis. Eur J Orthod 32: 582–588. 20660504 10.1093/ejo/cjp155

[41] Mostowska A, Biedziak B, Jagodzinski PP (2012) Novel MXS1 mutation in a family with autosomal-dominant hypodontia of second premolars and third molars. Arch Oral Biol 57: 790–795 doi:10.1016/j.archoralbio.2012.01.003 22297032 10.1016/j.archoralbio.2012.01.003

[42] Dietrich O (1932) Familienforschungen über die Zahnverhältnisse im oberen Schächental Will: Buchdruckerei Friedrich Gegenbauer. 40 p.

[43] Thomsen S (1952) Missing teeth with special reference to the population of Tristan de Cunha. Am J Phys Anthropol 10: 155–67. 14952565 10.1002/ajpa.1330100214

[44] Mahaney MC, Fujiwara TM, Morgan K (1990) Dental agenesis in the Dariusleut Hutterite Brethren: comparisons to selected Caucasoid population surveys. Am J Phys Anthropol 82: 165–177. 2360610 10.1002/ajpa.1330820205

[45] Polder BJ, Van’t Hof MA, Van den Linden PGM, Kuijpers-Jagtman AM (2004) A meta-analysis of the prevalence of dental agenesis of permanent teeth. Community Dent Oral Epidemiol 32: 217–226. 15151692 10.1111/j.1600-0528.2004.00158.x

[46] Bittles AH, Black ML (2010) Consanguineous marriage and human evolution. Ann Rev Anthropol 39: 193–207.

[47] Sholts SB, Clement AF, Wärmländer SKTS (2010) Brief communication: additional cases of maxillary canine-first premolar transposition in several prehistoric skeletal assemblages from the Santa Barbara Channel Islands of California. Am J Phys Anthropol 143: 155–160. 20564519 10.1002/ajpa.21343

[48] Bittles AH (2012) Consanguinity in context. Cambridge: Cambridge University Press.

[49] Gebel HGK (2004) Central to what? The centrality issue of the late PPNB mega-site phenomenon in Jordan. In: Bienert HD, Gebel HGK, Neef R, editors. Central settlements in neolithic Jordan. Studies in Early Near Eastern Production, Subsistence, and Environment 5. Berlin: Ex Oriente. 1–19.

[50] Benz M (2012) 'Little poor babies' – Creation of history through death at the transition from foraging to farming. In: Kienlin TL, Zimmermann A, editors. Beyond Elites. Universitätsforschungen zur Prähistorischen Archäologie 215. Bonn: Habelt. 169–182.

[51] Bentley RA, Bickle P, Fibiger L, Nowell GM, Dale CW, et al (2012) Community differentiation and kinship among Europe’s first farmers. PNAS 12, 109 (24): 9326–9330. 10.1073/pnas.1113710109 PMC3386065 22645332

[52] Bender F (1968) Geological map of Jordan, Sheet Aqaba-Ma’an. 1:250,000. Hannover: Bundesanstalt für Bodenforschung.

[53] Barjous MO (1988) Geological map sheet, Ash Shawbak (3151 III), 1: 50,000. Amman: Hashemite Kingdom of Jordan, Ministry of Energy and Mineral Resources, National Resource Authority, Geology Directorate.

[54] Barjous MO (1988) Geological map Petra & Wadi al-Lahyana (3050 I & 3050 IV), 1: 50,000. Amman: Hashemite Kingdom of Jordan, Ministry of Energy and Mineral Resources, National Resource Authority, Geology Directorate, Amman.

[55] Ibrahim KM, Rashdan M (1988) Geological map Wadi Gharandal (3050 III), 1: 50,000. Amman: Hashemite Kingdom of Jordan, Ministry of Energy and Mineral Resources, National Resource Authority, Geology Directorate.

[56] Kherfan AM (1998) Geological map of Bir Khidad, (3150 IV), 1: 50,000. Amman: Hashemite Kingdom of Jordan, Ministry of Energy and Mineral Resources, National Resource Authority, Geology Directorate.

[57] Rabba’ I (1991) Geological map of Al-Quarayqira, Jabal Hamra Faddan (3050 III), 1: 50,000. Amman: Hashemite Kingdom of Jordan, Ministry of Energy and Mineral Resources, National Resource Authority, Geology Directorate.

[58] Tarawneh K (2002) Geological map of Ma’an (3150 III), 1:50,000. Amman: Hashemite Kingdom of Jordan, Ministry of Energy and Mineral Resources, National Resource Authority, Geology Directorate.

