# Supplementary figures and images for: Earliest Evidence for Social Endogamy in the 9,000-Year-Old-Population of Basta, Jordan
Source: PLoS One. 2013 Jun 11;8(6):e65649. doi: 10.1371/journal.pone.0065649 (PMC3679157; doi:10.1371/journal.pone.0065649)

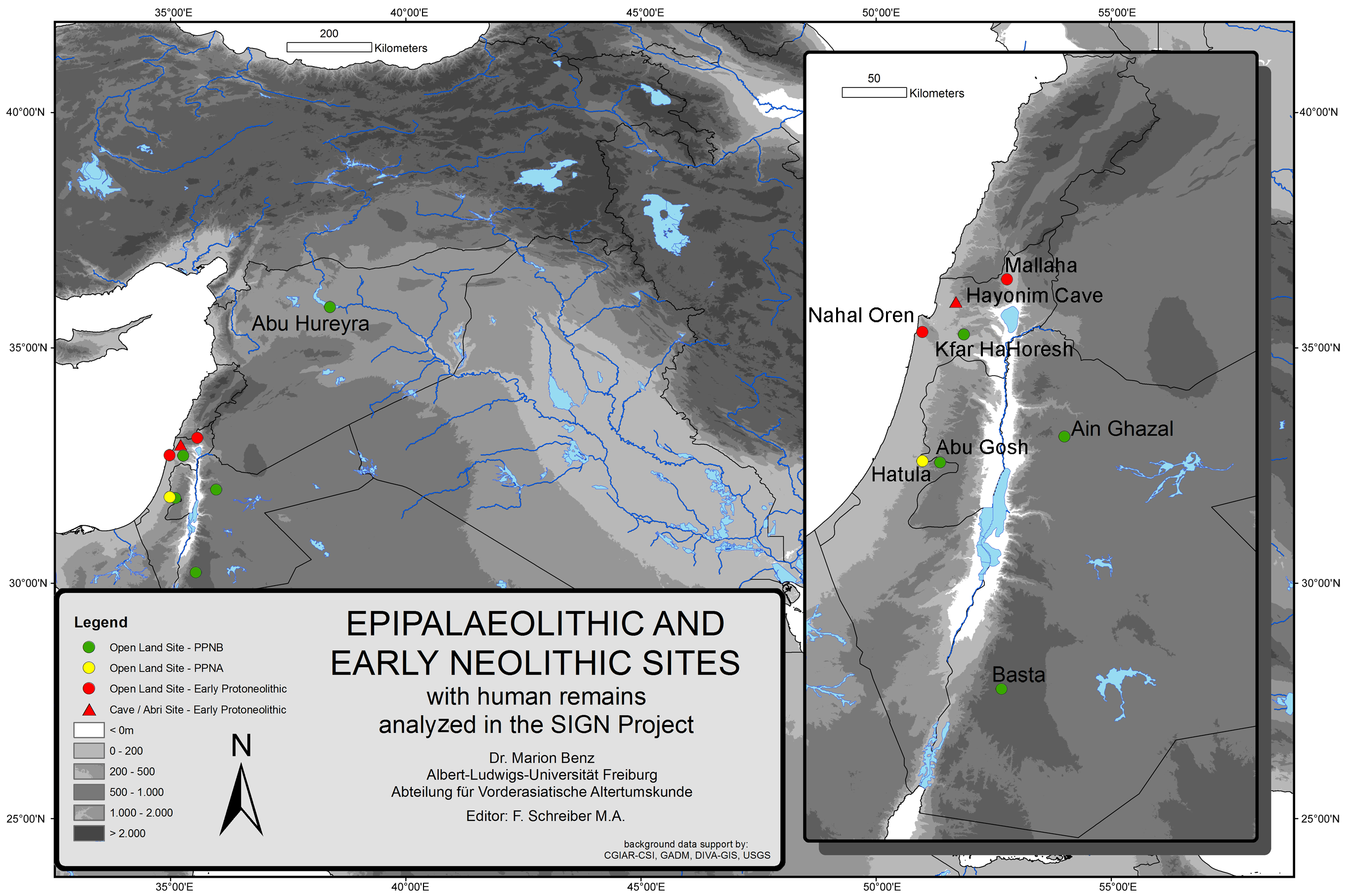

Supplement: Figure S1 — Map of the studied region. Location of Epipalaeolithic and early Neolithic sites with human remains investigated in the SIGN-Project (map edited by: Felix Schreiber). (TIF) [file pone.0065649.s001.tif]

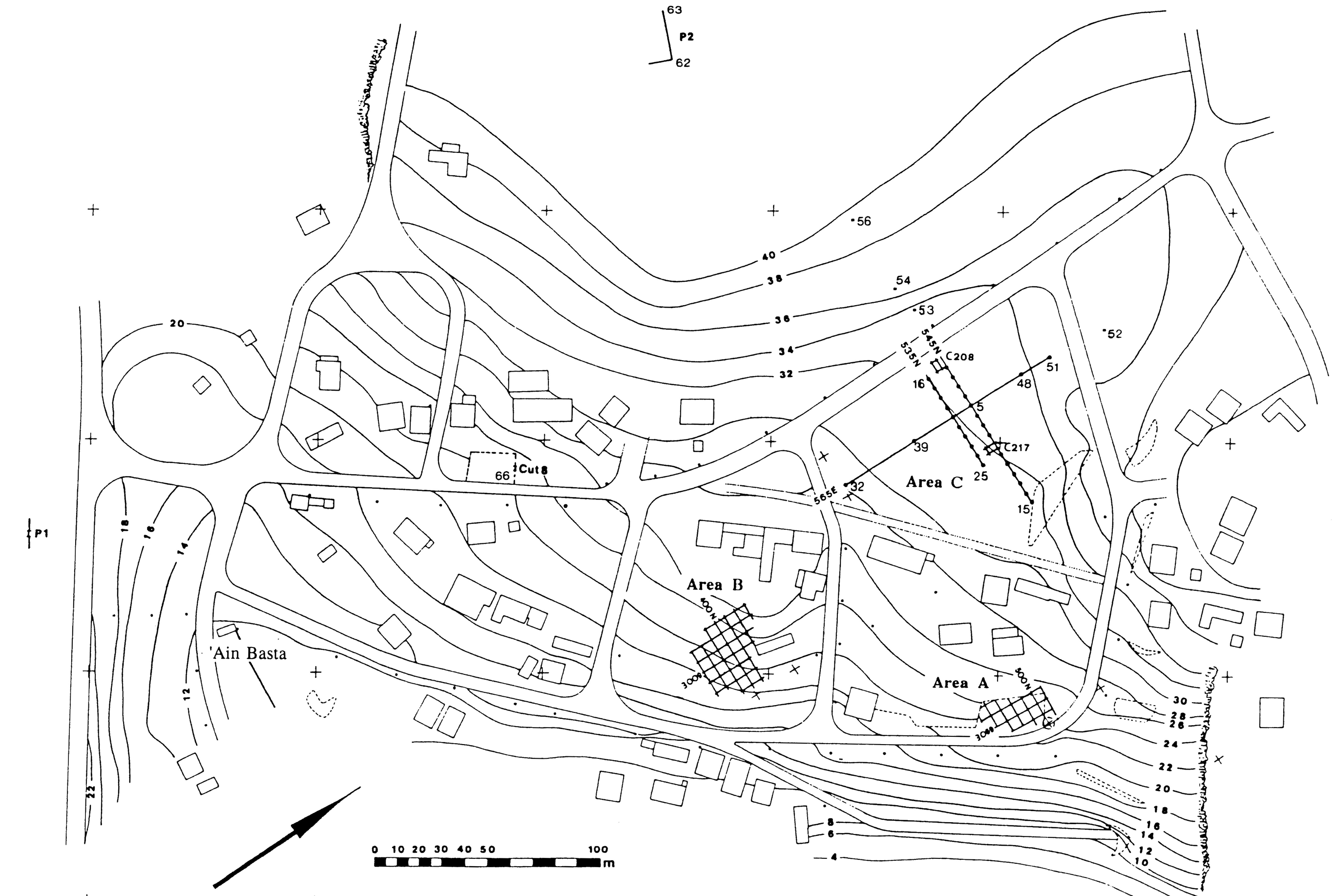

Supplement: Figure S2 — Plan of the modern Basta with the excavated Areas A, B, and C [21] . (TIF) [file pone.0065649.s002.tif]

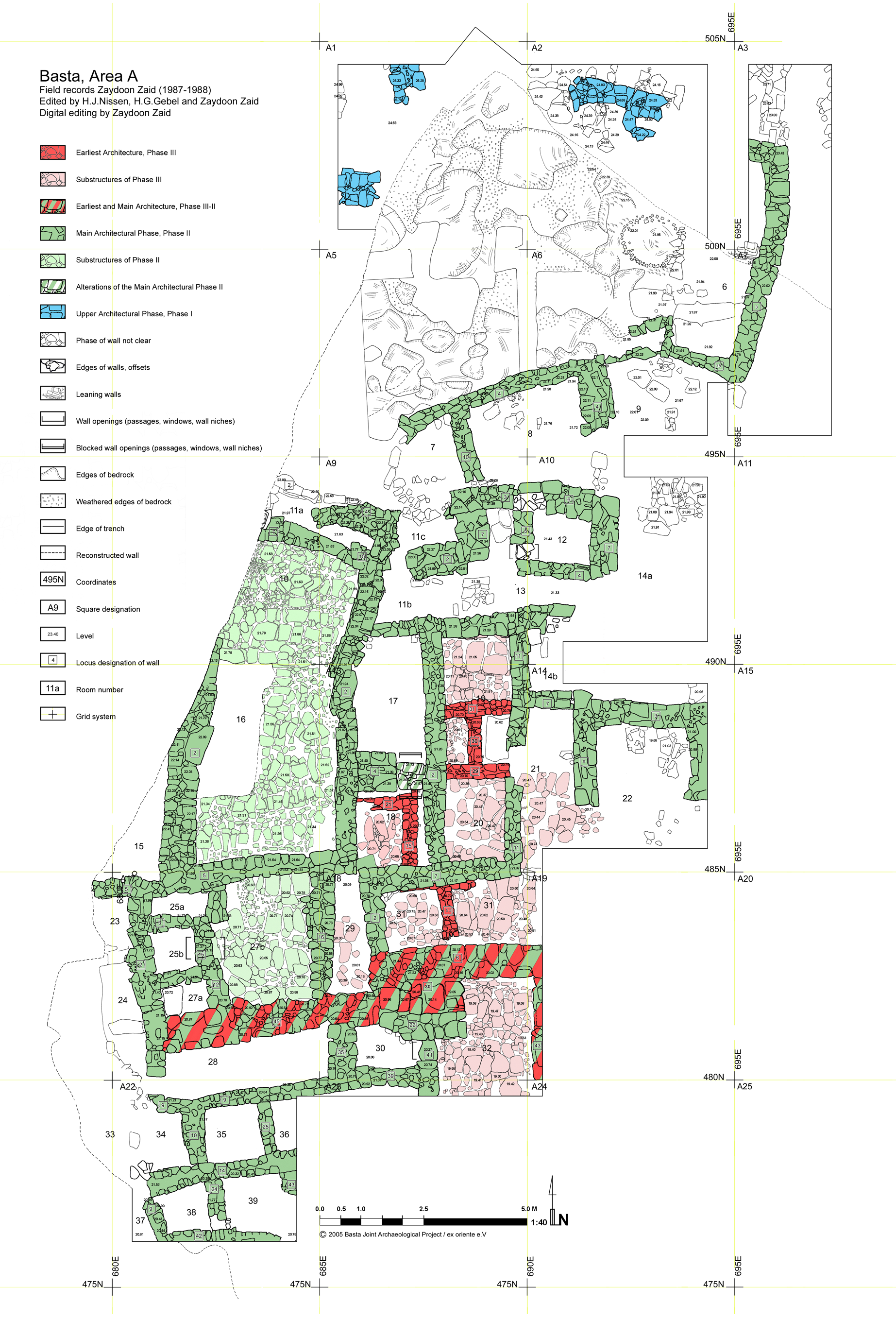

Supplement: Figure S3 — Area A. Architectural remains in Area A (Plan: Basta Joint Archaeological Project/ex oriente e.V.). (TIF) [file pone.0065649.s003.tif]

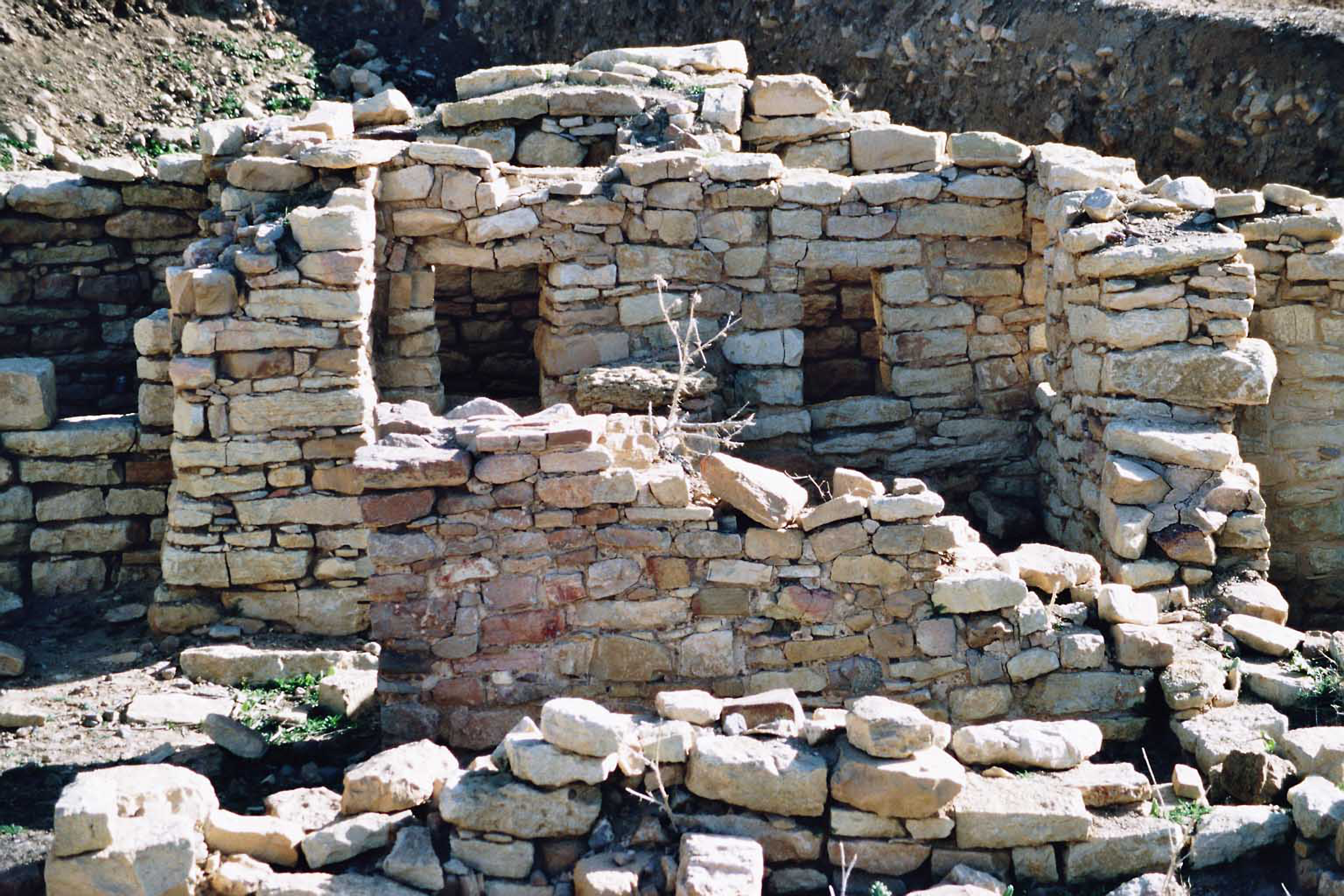

Supplement: Figure S4 — Area B. Preserved building remains in Area B ('Basta House') (Photo: M. Benz). (TIF) [file pone.0065649.s004.tif]

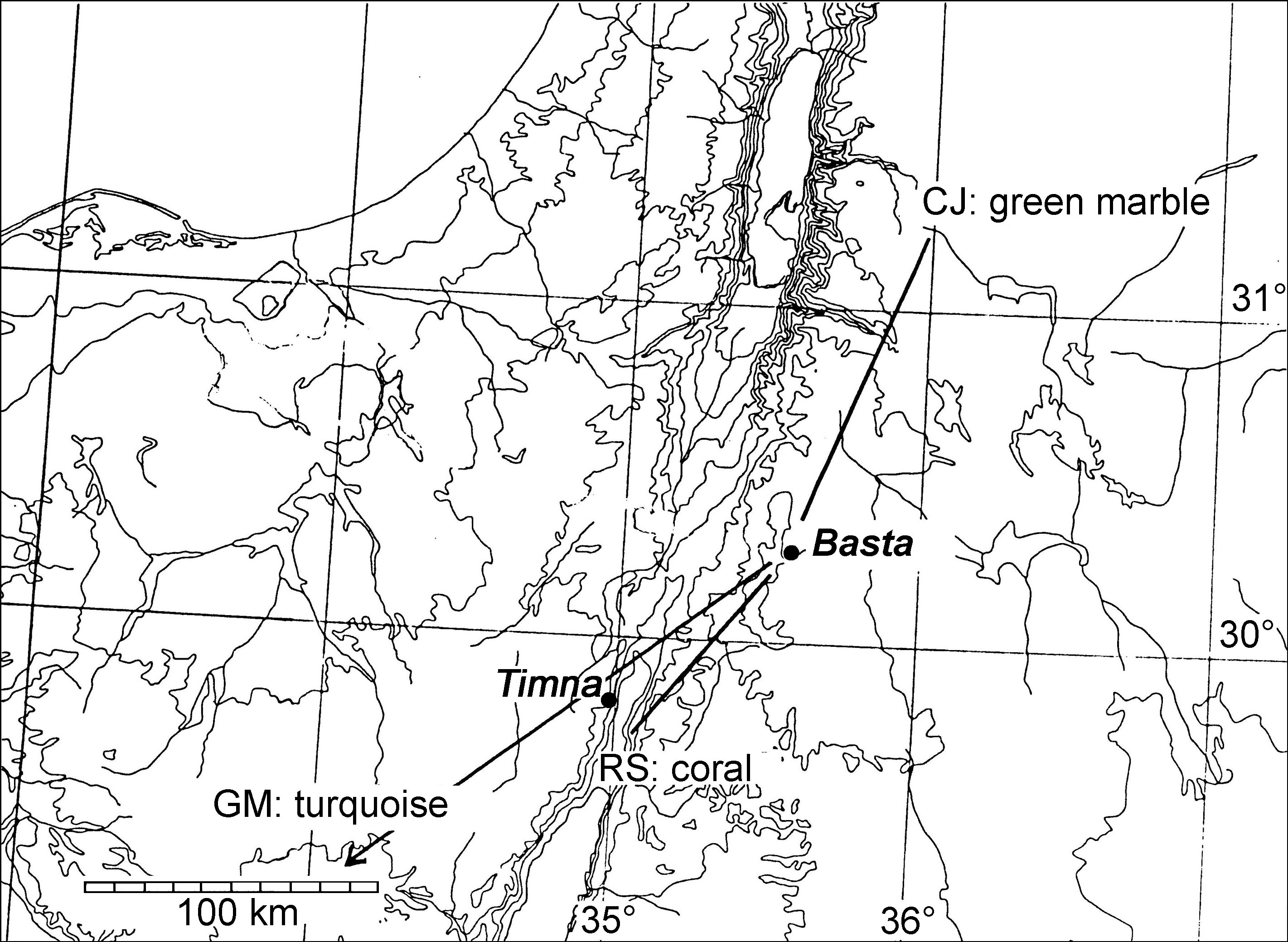

Supplement: Figure S5 — Raw material sources. Probable sources of raw materials which have been found at the early Neolithic site of Basta [17]. RS = Red Sea area, CJ = Daba, source area of green marble in Central Jordan, ∼50 km south of Amman, GM = Gebel el-Maghara, south-west Sinai (∼250 km southwest of Basta). (TIF) [file pone.0065649.s005.tif]

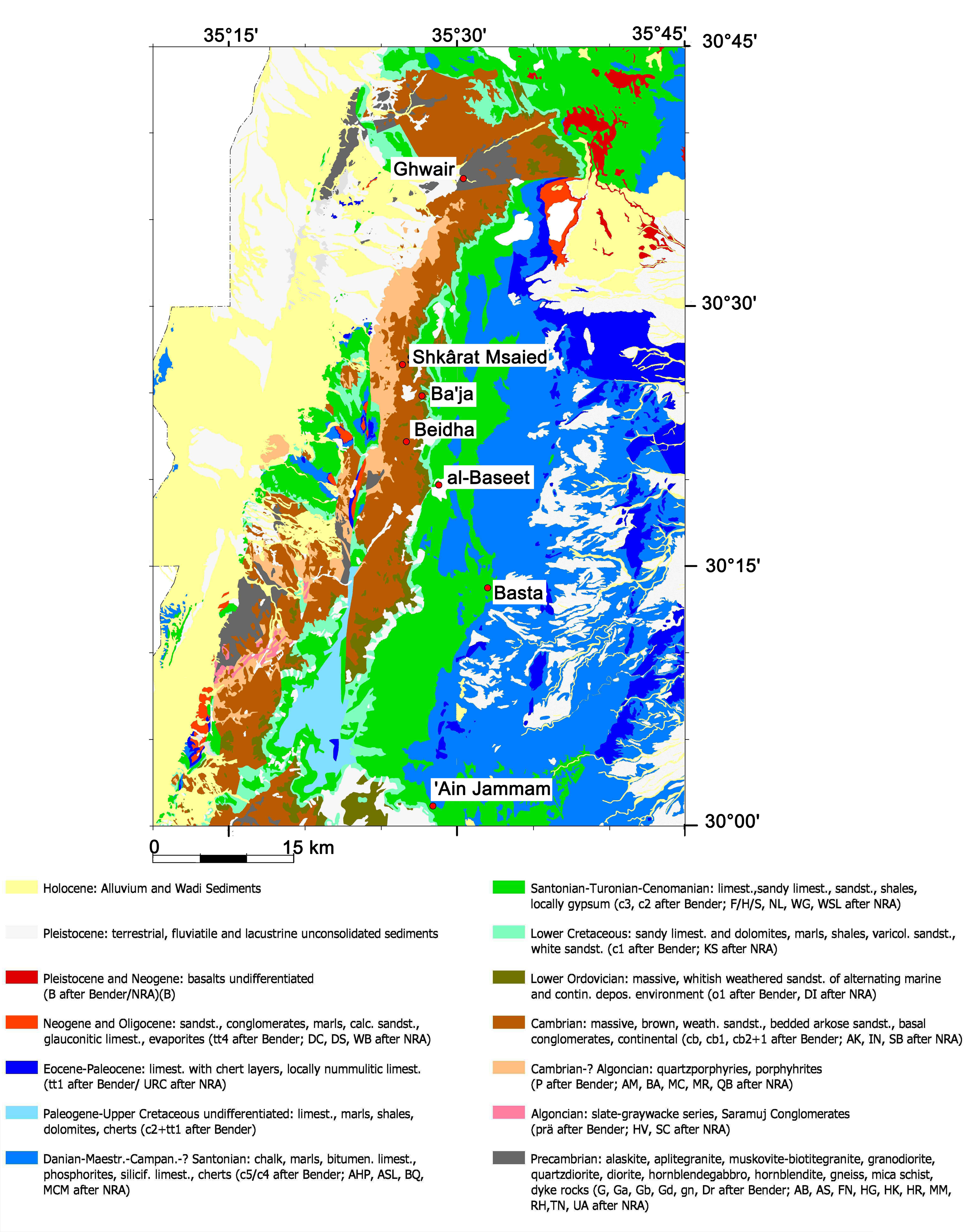

Supplement: Figure S6 — Geological setting of major Pre-Pottery Neolithic sites in southern Jordan. (Map design: Christoph Purschwitz, by compilation of data from [52]–[58]). (TIF) [file pone.0065649.s006.tif]
