# Supplementary material for: Earliest Evidence for Social Endogamy in the 9,000-Year-Old-Population of Basta, Jordan
Source: PLoS One. 2013 Jun 11;8(6):e65649. doi: 10.1371/journal.pone.0065649 (PMC3679157; doi:10.1371/journal.pone.0065649)
Supplement: Table S1 — Prevalence of maxillary lateral incisor agenesis (MLIA) within modern sampling groups. (DOC) [file pone.0065649.s007.doc]

**Table S1. Prevalence of maxillary lateral incisor agenesis (MLIA) within modern sampling groups.**

| **ID** | **Provenience** | **n** | **MLIA**  **(%)** | **MLIA + micro-dontia (%)** |
| --- | --- | --- | --- | --- |
| **I. General (dental) populations** | | | | |
| I.1 [62] | Jordan | 1005 | 2.8 | 5.1 |
| I.2 [63] | USA (Whites) | 500 | 2.2 | - |
| I.3 [64] | Pakistan | 2080 | 0.9 | - |
| I.4 [65] | Portugal | 16771 | 1.3 | 1.7 |
| I.5 [66] | Croatia | 568 | 1.8 | - |
| I.6 [67] | Germany | 10004 | 1.9 | 2.6 |
| I.7 [68] | Australia | 5127 | 2.2 | - |
| I.8 [69] | Israel | 21384 | 2.1 | - |
| I.9 [70] | Sweden | 1006 | 1.6 | - |
| I.10 [71] | Sweden | 5459 | 1.2 | - |
| I.11[72] | Iceland | 1116 | 0.9 | - |
| I.12[73] | Denmark | 3325 | 1.7 | - |
| I.13[74] | Malaysia | 1583 | 1.6 | - |
| I.14 [75] | Norway | 9532 | 1.2 | - |
| I.15 [76] | Japan | 8059 | 0.6 | 6.2 |
| I.16 [77] | S. Africa (Bushmen) | 189 | 0.5 | 6.8 |
| **II. Selected (dental) populations** | | | | |
| II.1 [78] | Norway | 1953 | 2.0 | - |
| II.2 [79] | Slovenia | 212 | 6.4 | - |
| II.3 [80] | USA | 1000 | 1.1 | 1.9 |
| II.4 [81] | Kenya | 615 | 2.7 | - |
| II.5 [82] | Germany | 1552 | 1.6 | - |
| II.6 [83] | Turkey | 2761 | 3.2 | - |
| II.7 [84] | Italy | 241 | 2.5 | - |
| II.8 [85] | Mexico | 668 | 5.5 | - |
| II.9 [86] | Korea | 1622 | 2.2 | - |
| II.10[36] | USA (control families) | 918 | 2.8 | - |
| **III. Parents and siblings** | | | | |
| III.1 [69] | Israel | 426 | 10.4 |  |
| III.2 [39] | Portugal | 142 | 17.4 |  |
| III.3 [70] | Sweden | 1006 | 29.0 | - |
| III.4 [36] | USA | 487 | 17.7 | - |
| **IV. Geographically isolated populations** | | | | |
| IV. 1[42] | Switzerland (Alpine valley) | 450 | 1.6 | 2.4 |
| IV.2 [30] | Finland (Island Hailuoto) | 306 | 4.3 | 5.6 |
| IV.3 [43] | Tristan da Cunha (Atlantic Ocean; Island population) | 188 | 0.7 | 5.0 |
| IV.4 [14] | Switzerland (Alpine valley) | 162 | 21.0 | 33.3 |

**References of Table S1**

1. Albashaireh ZS, Khader YS (2006) The prevalence and pattern of hypodontia of the permanent teeth and crown size and shape deformity affecting upper lateral incisors in a sample of Jordanian dental patients. Community Dent Health 23: 239-243.
2. Hrdlicka A (1921) Further studies of tooth morphology. Am J Phys Anthropol 4: 141-146.
3. Malik SA (1972) Missing and rudimentary upper lateral incisors: a statistical survey. J Dent 1: 25-27.
4. Pinho T, Tavares P, Maciel P, Pollmann C (2005) Developmental absence of maxillary lateral incisors in the Portuguese population. Eur J Orthod 27: 443-449.
5. Prskalo K, Zjaca K, Skarić-Jurić T, Nikolić I, Anić-Milosević S, et al. (2008) The prevalence of lateral incisor hypodontia and canine impaction in Croatian population. Coll Antropol 32: 1105-1109.
6. Riemschneider K (1963) Über die Häufigkeit von Nichtanlagen und Kümmerformen sowie die Durchschnittsbreite bleibender oberer seitlicher Schneidezähne bei 10,000 Rostocker Schulkindern. Dtsch Stomatol 13: 202-214.
7. Symons AL, Stritzel F, Stamation J (1993) Anomalies associated with hypodontia of the permanent lateral incisor and second premolar. J Clin Pediatr Dent 17: 109-111.
8. Chosack A, Eidelman E, Cohen T (1975) Hypdontia: a polygenic trait – a family study among Israeli Jews. J Dent Res 54: 16-19.
9. Grahnén H (1956) Hypodontia in the permanent dentition. A clinical and genetic investigation. Odontol Revy 7, Suppl. 3: 1-100.
10. Thilander B, Myberg N (1973) The prevalence of malocclusion in Swedish schoolchildren. Scand J Dent Res 81: 12-21.
11. Magnusson TE (1977) Prevalence of hypodontia and malformations of permanent teeth in Iceland. Community Dent Oral Epidemiol 5: 173-178.
12. Rolling S (1980) Hypodontia of permanent teeth in Danish schoolchildren. J Dent Res 88: 365-369.
13. Nik-Hussein NN (1989) Hypodontia in the permanent dentition: a study of its prevalence in Malaysian children. Aust Orthod J 11: 218-220.
14. Nordgarden H, Jensen JL, Storhaug K (2001) Reported prevalence of congenitally missing teeth in two Norwegian counties. Community Dent Health 19: 258-261.
15. Sumiya Y (1959) Statistic study on dental anomalies in the Japanese. J Anthropol Soc Nippon 67: 215-233.
16. Cleaton-Jones P (1970) Agenesis and peg-shaped permanent maxillary lateral incisors in Kalahari Bushmen. J Dent Res 49: 457.
17. Aasheim B, Øgaard B (1993) Hypodontia in 9-year-old Norwegian related to need of orthodontic treatment. Scand J Dent Res 101: 257-260.
18. Fekonja A (2005) Hypodontia in orthodontically treated children. Eur J Orthod 27: 457-460.
19. Horowitz JM (1966) Aplasia and malocclusion: a survey and appraisal. Am J Orthod 52: 440-453.
20. Ng’ang’a RN, Ng’ang’a PM (2001) Hypodontia of permanent teeth in a Kenyan population. East Afr Med J 78: 200-203.
21. Roth P, Hirschfelder U (1990) Hypodontia with anlage of all four third molars. Dtsch Zahnarztl Z 45: 267-269.
22. Topkara A, Sari Z (2011) Prevalence and distribution of hypodontia in a Turkish orthodontic patient population: results from a large academic cohort. Eur J Paediatr Dent 12: 123-127.
23. Galluccio G, Pilotto A (2008) Genetics of dental agenesis: anterior and posterior area of the arch. Eur Arch Paediatr Dent 9: 41-45.
24. Silva Meza R (2003) Radiographic assessment of congenitally missing teeth in orthodontic patients. Int J Paediatr Dent 13: 112-116.
25. Chung CJ, Han JH, Kim KH (2008) The pattern and prevalence of hypodontia in Koreans. Oral Dis 14: 620-625.
