# Supplementary material for: Earliest Evidence for Social Endogamy in the 9,000-Year-Old-Population of Basta, Jordan
Source: PLoS One. 2013 Jun 11;8(6):e65649. doi: 10.1371/journal.pone.0065649 (PMC3679157; doi:10.1371/journal.pone.0065649)
Supplement: Text S1 — Supplementary text. (DOC) [file pone.0065649.s008.doc]

Supplementary Text

Social Identities of Early Neolithic Groups in the Near East (SIGN-Project)

The importance of social processes in relationship to economic and ecological changes at the transition from foraging to farming has long been underestimated. However, ethnographic observations demonstrate that the generalized reciprocity, the principle of sharing and open access to resources and land, had to be reduced to a circumscribed group before regular farming and herding could be permanently established [59]. The main aim of the project “Social Identities of Early Neolithic Groups in the Near East” (SIGN) was, therefore, to reassess this ethnographic observation based on the archaeological data and if so, to clarify on which criteria these groups were defined.

This was tested on three levels: Firstly, symbolic and ritual expressions of different groups in the Near East [60], secondly, on the flint technology and technological transfer networks [61], and thirdly, on the biological anthropological level to gain information about familial relationships within and among the different groups. Chronological and geographic information on all investigated sites were assembled in an open access database for radiocarbon dates and geographical position of the sites, in order to discern whether changes in burial customs and technological remains were due to chronological or geographical differences [16].

The study area comprises the countries of the Fertile Crescent (Jordan, Israel, Palestine, Lebanon, Syria, southeastern Turkey, northern Iraq, and western Iran). Anthropological remains of nine sites from the southern Levant to the middle Euphrates could be analyzed for their epigenetic markers on teeth (Figure. S1). These are Hayonim Cave, Nahal Oren and Mallaha for the late Epipalaeolithic (Natufian: 12800-9700 cal BC), the Pre-Pottery Neolithic A site of Hatula (10150-9320 cal BC) as well as Abu Gosh, Kfar HaHoresh, Abu Hureyra, 'Ain Ghazal and Basta for the middle and late Pre-Pottery Neolithic B (8300-7000 cal BC). The anthropological sub-project used a non-invasive method [26] to detect familial relationships on the basis of epigenetic characteristics of dental and skull remains. Another part of the anthropological sub-project was to reconstruct possible migration patterns by strontium and oxygen analyses on tooth enamel of selected individuals.

**References Text S1**

1. Benz M (2000) Die Neolithisierung im Vorderen Orient. Theorien, archäologische Daten und ein ethnologisches Modell. Studies of Early Near Eastern Production, Subsistence, and Environment 7. Berlin: Ex Oriente. 260 p.
2. Benz M (2010) Beyond death – the construction of social identities at the transition from foraging to farming. In: Benz M, editor. The principle of sharing. Segregation and construction of social identities at the transition from foraging to farming. Studies in Early Near Eastern Production, Subsistence, and Environment 14. Berlin: Ex Oriente. pp. 249-276.
3. Ali N (2010) Style, society and lithic production during the Late Natufian and Early Neolithic periods in the southern Levant. In: Benz M, editor. The principle of sharing. Segregation and construction of social identities at the transition from foraging to farming. Studies in Early Near Eastern Production, Subsistence, and Environment 14. Berlin: Ex Oriente. pp. 223-248.
